# *Icacina senegalensis *(Icacinaceae), traditionally used for the treatment of malaria, inhibits *in vitro Plasmodium falciparum *growth without host cell toxicity

**DOI:** 10.1186/1475-2875-10-85

**Published:** 2011-04-11

**Authors:** Serigne O Sarr, Sylvie Perrotey, Ibrahima Fall, Saïd Ennahar, Minjie Zhao, Yérim M Diop, Ermanno Candolfi, Eric Marchioni

**Affiliations:** 1 Equipe de Chimie Analytique des Molécules BioActives IPHC-LC4, UMR 7178, Faculté de Pharmacie, 74, route du Rhin, 67400, Illkirch, France; 2 Laboratoire de Chimie Analytique et Bromatologie, Faculté de Médecine et de Pharmacie, Université Cheikh Anta DIOP, B.P 5005, Dakar-Fann, Sénégal; 3 Institut de Parasitologie et de Pathologie Tropicale, Université de Strasbourg, EA 4438, 3 rue Koeberlé, 67000 Strasbourg, France; 4 Laboratoire de Pharmacognosie et Botanique, Faculté de Médecine et de Pharmacie, Université Cheikh Anta DIOP, B.P 5005, Dakar-Fann, Sénégal

## Abstract

**Background:**

With the aim of discovering new natural active extracts against malaria parasites, *Icacina senegalensis *was selected after an ethnopharmacological survey conducted on plants used in traditional malaria treatment in Senegal.

**Methods:**

Different concentrations of the plant extract and fractions were tested on synchronized *Plasmodium falciparum *cultures at the ring stage using the parasite lactate dehydrogenase assay. Their haemolytic activity and *in vitro *cytoxicity were evaluated. The chromatographic profiles of active fractions were also established.

**Results:**

The plant extract and fractions revealed anti-plasmodial activity (IC_50 _< 5 μg/mL) with no toxicity (Selectivity indexes >10). The dichloromethane fraction showed stronger anti-plasmodial activity than the total extract.

**Conclusion:**

Anti-plasmodial activity and toxicity of *I. senegalensis *are reported for the first time and showed promising results in malaria field research.

## Background

Despite intensive efforts to control malaria, the disease continues to be one of the greatest health problems facing Africa [1]. Then the global scope of malaria and the spread of drug-resistant *Plasmodium falciparum *make the need for improved therapy undeniable. There were an estimated 247 million malaria cases among 3.3 billion people at risk in 2006, causing nearly one million deaths, mostly children under five years of age. One hundred six countries were endemic for malaria in 2009, 45 within the WHO African region [1].

This worsening situation can be explained by resistance of *P. falciparum *to the current anti-malarial drugs [2], lack of new therapeutic targets, unaffordability and poor quality of anti-malarial drugs [3,4] and their bad storage under tropical conditions [4]. Then an urgent need for the development of new anti-malarial agents faces the scientific community.

Traditional medical knowledge based on the use of natural products from plants has often been the basis for discovering new drugs. It is estimated that 80% of many developing countries population still use plant-based traditional medicines [5]. These natural products and their derivatives represent almost half of the drugs approved since 1994 [6] and more than 30% of the current anti-malarial market [7].

In this context, an ethnopharmacological survey of medicinal plants was carried out in Senegal and data were collected on some plants traditionally used for the treatment of malaria and fevers. Moreover, a literature review was conducted and allowed the identification of *Icacina senegalensis *A. Juss., a plant with known traditional medical applications but up to now with no reported anti-plasmodial activity in scientific literature. Then this plant was investigated in this work for its anti-plasmodial activity.

Until recently, schizont maturation inhibition technique [8] and isotopic *in vitro *assays were the main methods used to assess *in vitro *susceptibility of *P. falciparum *to anti-plasmodial drugs. Their use was however limited by the technical constraints of reproducibility of schizont production and radioactive risk management. This explains the revived interest in the development of replacement methods, such as the detection of malaria antigen. The sensitive quantification of major parasite proteins (Histidine Rich Protein 2, Lactate Dehydrogenase, Aldolase) by sandwich enzyme-linked immunosorbent assay (ELISA) has recently gained particular interest [9,10]. In this study *Plasmodium *lactate dehydrogenase (pLDH) immunodetection assay was used [11].

This is the first scientific demonstration of the anti-plasmodial activity of *I. senegalensis *leaf extracts in a standard *in vitro *assay based on pLDH detection. The cytotoxicity of extracts was also evaluated.

## Methods

### Ethnopharmacological survey

During an ethnopharmacological survey carried out between July to December 2007 in the province of Médina Sabakh (Western-Center of Senegal), based on a questionnaire, ten healers and members of the local population were interviewed to determine on which symptoms their diagnosis/feeling of suspected malaria was based, which parts of plants were used to prepare the traditional treatment for the suspected malaria, how it was administrated, and finally whether these traditional treatments were effective on the observed symptoms.

Following this investigation, the traditional diagnose of suspected malaria was based on observation of fever, strong headache, chills, vomits, loss of apetite, increased fatigue. These symptoms were observed in particular during the rainy season in Senegal (June to October). The fever was the most known symptom for a suspected malaria. For better efficiency, the traditional treatment was supposed to be administered at the onset of clinical symptoms.

The survey revealed seven plants traditionally used for malaria treatment in Senegal: *Azadirachta indica *(leaves and stem bark), *Khaya senegalensis *(stem bark), *Anogeissus leiocarpus *(stem bark), *Ficus gnaphalocarpa *(stem bark), *I. senegalensis *(leaves), *Nauclea latifolia *(leaves, stem bark and roots), *Cassia occidentalis *(leaves). A literature review (ScienceDirect, PubMed, Napralert, SciFinder) indicated that among the plants used in traditional malaria treatment in Senegal and revealed by this survey, *I. senegalensis *had until today not been studied for its anti-plasmodial activity. All ten traditional healers interviewed revealed that they used *Icacina *leaves powder to treat successfully "malaria". This plant was also well known by the local population.

### Selection and collection of plant materials

**Figure 1:**
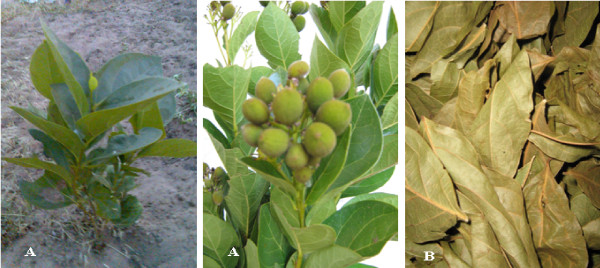
***Icacina senegalensis *at different development stages (A) and dried leaves (B)**.

**Figure 2:**
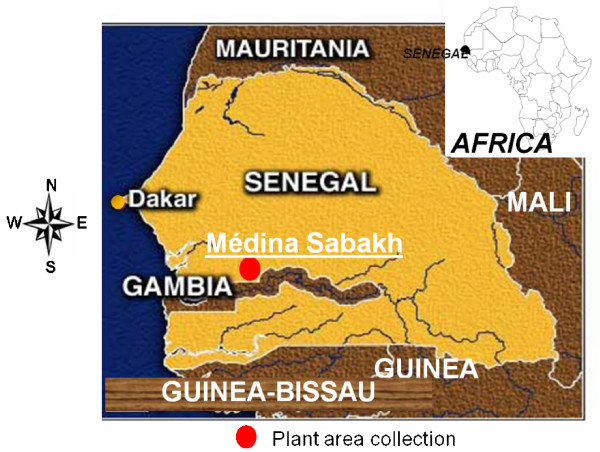
**Map of Senegal showing plant area collection**.

*Icacina senegalensis *(Icacinaceae) leaves (Figure 1A) used against malaria and symptoms that can be possibly related to malaria, were harvested in October 2007 in the plant's natural habitat in the province of Médina Sabakh. This area is in 274 km distance from the capital Dakar and lies within the geographical coordinates of 15°34'W and 13°35'N (Figure 2). The leaves were collected early in the morning before sun set as the local population who take decoction and maceration of powdered leaves usually do. After identification at the Dakar's Laboratoire de Pharmacognosie et Botanique (M. Fall Ibrahima and Dr William Diatta), a voucher specimen SS 005 was deposited at the herbarium of the Faculté de Médecine, Pharmacie and Odonto-Stomatologie of Dakar. This plant is not featured in the World Conservation Union's (IUCN) red list of threatened species and its harvest is not prohibited (IUCN red list).

### Plants extraction, extracts partition and fractionation

The plant material (5 kg) consisted of leaves of *I. senegalensis *without stems. The leaves were air-dried at room temperature (approximately 30°C) on a laboratory bench, safe from the light during ten days (Figure 1B). They were cut out and ground with a knife crusher (Illico, Moulinex, France), then, in a grinder Sprex SamplePrep 6870 Freezer Mill cryogenic (Sprex, Metuchen, New Jersey, USA).

Methanol was used as polar solvent extraction. Forty grams of the powdered plant material (0.2 mm) was submitted to three successive 90-min Soxhlet extractions (Isolab, Wertheim, Germany) with analytical grade methanol (120 mL). The three organic extracts were pooled. The methanolic extracts named IM were evaporated to dryness under vacuum (35°C, 150 mbar) (Rotavapor, Büchi, Sweden). As some moisture remained in the powder, it was resuspended in 10 mL of purified milliQ+ water (Millipore, Molsheim, France), transferred in lyophilization vessels, freeze dried (Alpha 2-4 LSC/Bioblock Scientific, Strasbourg, France) and stored at +4°C safe from light.

The freeze-dried methanolic extract (IM) was redissolved in 100 mL water/methanol (20/80; v/v) by sonication (Sonorex RK100, Bandelin Electronic, Berlin, Germany) during 30 min and then partitioned with 3 × 40 mL pentane. The pentane fraction (IMP) was discarded whereas the water one was partitioned with 3 × 40 mL dichloromethane to give apolar (IMD) and polar fractions (IMW) which were both freeze dried and stored under safe light conditions at +4°C before testing. Finally the three fractions named IM, IMD and IMW were tested for their anti-plasmodial activity on *P. falciparum *3D7 Africa and *P. falciparum *7G8 Brazil strains respectively chloroquine-sensitive and chloroquine-resistant.

The most active fraction (IMD) (200 mg) was separated on Sephadex LH-20 ^®^(GE Health care, Uppsala, Sweden) column (Length = 32 cm, Internal Diameter = 2.5 cm) with methanol/water (80:20; v/v) (1 L) as eluent. The subfractions (10 mL each) obtained were screened by means of high performance liquid chromatography (HPLC) system with diode array detection (DAD) (Acquity, Waters corporation, MA, USA). The chromatographic separations were performed on a Kinetex^® ^C18 column (2.6 *μ*m, 2.1 × 150 mm) (Phenomenex, Le Pecq, France) maintained at 30°C and eluted with a linear methanol-aqueous formic acid (0.11%; v/v) gradient up to 19 min at 250 μL/min, starting at 10% methanol and rising to 80% over the course of the gradient. A volume of 2.5 μL of IMD fractions (100 ppm) was injected. Only analytical grade solvents (HPLC-quality) and reagents were used. The DAD conditions were: 320 nm, Max plot or spectra 200-500 nm, resolution at 1.2 nm, sampling rate at 20 points/sec (pps, Hz).

Subfractions with similar chromatographic profile were then pooled and gave three fractions Da (subfractions 1 to 10), Db (subfractions 11 to 25) and Dc (subfractions 26 to 100) which were also tested for cytotoxicity and *in vitro *activity on both 3D7 and 7G8 *P. falciparum *strains.

### Preparation of samples and controls

Samples and controls are prepared identically with 0.5% dimethylsulfoxyde (DMSO) at maximal final concentration in Malaria Culture Medium (MCM). It was a modification of that described in a previous culture method [12] and consisted of RPMI 1640 with glutamax^® ^(Gibco, Cergy-Pontoise, France) supplemented with 10% (v/v) foetal calf serum, 25 mM HEPES, 1 μg/mL hypoxanthin, 0.11 mg/mL sodium pyruvate and 0.02 mg/mL gentamicin. Stock solutions of plant extracts at 1 mg/mL in MCM were used to prepare the test samples. When necessary, solubilization of samples was assisted by sonication (Sonorex RK100, Bandelin Electronic, Berlin, Germany).

For anti-plasmodial test, two negative controls were MCM and a freeze-dried methanolic extract of *Khaya senegalensis *stem bark prepared identically as IM from plants harvested in Senegal while chloroquine was the positive control. Concerning the plant used as negative control, a previous study showed that *Khaya senegalensis *stem bark methanolic extract gave an IC_50 _on 3D7 of 150 μg/mL while butanolic and aqueous extracts gave respectively IC_50s _of 500 and 100 μg/mL on *P. falciparum *Dd2, a chloroquine-resistant strain [13]. Also the ethanolic extract of *K. senegalensis *gave IC_50 _of 82 μg/mL on fresh clinical isolates of *P. falciparum *[14]. These results justify the use of this plant extract as negative control besides the extracts dissolution solvents, which showed no effect on parasite growth.

### Parasite strains and *in vitro *culture

Plants extracts were tested on strains of *P. falciparum *3D7 Africa (chloroquine-sensitive) and 7G8 Brazil (chloroquine-resistant) (Laboratoire de Parasitologie, Centre National de Référence du Paludisme, Hôpital Bichat-Claude Bernard, and Université Paris Descartes, EA 209, Paris, France).

Uninfected human blood group O+ erythrocytes used as host cells were obtained from healthy donors (Etablissement Français du Sang, Strasbourg, France) and conserved in SAGM (Saline Adenine Glucose Medium) at 37.5% (v/v) (Sigma Aldrich, St Quentin Fallavier, France).

This preparation was washed with RPMI 1640 medium before every use and the stock solution prepared at a 50% haematocrit. The strains were routinely maintained in continuous long-term cultures at the Institut de Parasitologie et de Pathologie Tropicale (Strasbourg, France) at 37°C in an atmosphere of 3% O_2_, 5% CO_2 _and 91% N_2 _(Sanyo O_2_/CO_2 _incubator, model MCO-5M, Avon, France). The parasite density was maintained at 1% parasitaemia. The parasitaemia is calculated as being the number of erythrocytes parasitized when observing 10 microscopic fields (Zeiss, Axioskop, Germany). Before each anti-plasmodial test, parasite cultures were synchronized in a ring stage obtained from serial treatment with 5% D-sorbitol [15].

### Haemolytic activity

It was done according to a modified method previously described [16]. Briefly, the extracts were serially diluted in MCM at concentrations ranging from 1 to 100 μg/mL in 96-well culture plates and each concentration incubated with the same volume (100 μL) of non-infected erythrocytes (5% haematocrit). After 40 min of incubation at 37°C under circular agitation (Titramax 100 Heidolph, Schwabach, Germany), erythrocytes were sedimented in the microtitre plate wells by centrifugation (400 g for 7 min) (Fisher Bioblock Scientific, Illkirch, France). The supernatants were diluted 1:4 in distilled ultra pure water in separate microtitre plates. An haemolytic agent, 5% Sodium Dodecyl Sulfate (SDS), was used as positive control. Negative control contained erythrocytes diluted (v/v) with the sterile MCM. Haemolytic effect of chloroquine was also evaluated. Haemoglobin content in the supernatants was determined by absorbance measurements at 538 nm in a microtiter plate spectrophotometer (ELX 808, Bio-Tek, Winooski, USA).

### Cytotoxicity assay

The cytotoxicity was assessed in culture on hepatic mouse cells Hepa 1-6 obtained from the American Type Culture Collection (ATCC^® ^Number: CRL-1830, Manassas, USA) and on Normal Human Dermal Fibroblasts (NHDF) cells (PromoCell, Heidelberg, Germany). The assessment was performed in order to determine selectivity indexes (SI) which represents the ratio of cytotoxicity to anti-plasmodial activity.

To estimate the half max inhibitory concentration for cytotoxicity (IC_50_), the 3-(4,5-dimethylthiazol-2-yl)-2,5-diphenyltetrazolium bromide (MTT) (Sigma Aldrich, St Quentin Fallavier, France) enzymatic micromethod was used [17]. Hepa 1-6 cells were cultured in suspension in complete Dulbecco's Modified Eagle Medium (DMEM) containing 10% heat-inactivated fetal calf (FCS) (alpha-calf) in the presence of 5% CO_2 _at 37°C. Medium was renewed at 2-days intervals. NHDF cells were cultured in Fibroblast Growth Medium Kit (PromoCell, Heidelberg, Germany) in the presence of 5% CO_2 _at 37°C.

Cells were harvested and washed by centrifuging for 10 min at 400 × g, then counted and adjusted to a final concentration of 5 × 10^4 ^cells/well in 24-well-flat-bottom microplates (Nunc). Hundred microlitres of culture medium were added to each well containing cells. Plant extracts were dissolved in DMSO (with a final concentration not exceeding 0.5%) to prepare stock solutions at 1 mg/mL. The stock solutions were diluted with the culture medium to obtain the desired test concentrations. Then, 100 μL of each extract solution was added to cells at fifteen different concentrations ranging from 1 to 500 μg/mL. Cells were incubated at 37°C with 5% CO_2_. After 24 h of incubation, the supernatant was removed and 100 μL of MTT (0.5 mg/mL) was added to each well [18]. Plates were further incubated for 3 h. The enzymatic reaction was then stopped by addition of 100 μL of a mixture of ethanol-DMSO (50:50; v/v). The plates were incubated for an additional 30 min under agitation at room temperature. The solution was transferred into a 96-well plate to measure the absorbance at 570 nm using a microtiter plate spectrophotometer (ELX 808, Bio-Tek, Winooski, USA). Cells cultivated in absence of treatment but maintained under the same conditions were used as control. Cytotoxicity of chloroquine was also tested on cells.

### *In vitro *anti-plasmodial assay

Parasites in culture medium were used as a negative control and represented 100% parasite viability. The positive controls consisted of chloroquine diphosphate (Sigma-Aldrich, Saint Quentin Fallavier, France), at concentrations varying between 0 and 100 nM for *P. falciparum *3D7 strain. For *P. falciparum *7G8 strain, tested chloroquine concentrations reached 1.5 μM.

Extract testing was performed three times in triplicate in a 96-well culture plate with cultures mostly at ring stages at 1% parasitaemia (haematocrit, 2%). Parasite culture (130 μL) was incubated with each extract (130 μL) for 96 h at 37°C in an atmosphere of 3% O_2_, 5% CO_2 _and 91% N_2 _(Sanyo O_2_/CO_2 _incubator, model MCO-5M, Avon, France). All reagents and buffers used for the pLDH test were from Diamed (DiaMed, Cressier s/Morat, Switzerland). The anti-malarial activity of plant extracts was evaluated by the pLDH immunodetection assay with a commercially available sandwich enzyme-linked immunosorbent assay following manufacturer recommendations. Briefly, the test contains ELISA plates already coated with a monoclonal antibody (MAb) against pan-*p*LDH. After addition of lysis buffer (100 μL), 50 μL of the incubated parasitized supernatant culture and 50 μL of pLDH controls (positive and negative), the ELISA plates were incubated for 1 h at 37°C under soft shaking and washed with phosphate buffer. After the addition of 100 μL per well of a biotinylated MAb against pan-*p*LDH, the plates were incubated for 30 min at 37°C and then washed again with phosphate buffer. A third incubation for 15 min at 37°C with 100 μL of a streptavidin horse radish peroxidase solution was followed by a last washing step (phosphate buffer). Enzyme activity was revealed by incubation for 15 min at 37°C with 100 μL of tetramethylbenzidine. The reaction was stopped with 50 μl of 0.5 M sulfuric acid and the absorbance was read with a microplate spectrophotometer (ELX 808; Bio-Tek, Winooski, USA) at 450 nm [9,19]. The absorbance values from negative control wells (containing only MCM and the culture) were referred to as having 100% pLDH activity.

### Selectivity indexes

The selectivity index is defined as the ratio of the IC_50 _value determined on the NHDF cells (cytotoxicity) on the IC_50 _value determined on *P. falciparum *3D7 (anti-plasmodial activity).

### Data analysis

All results included IC_50 _(concentration at which the parasite growth was inhibited by 50%) are reported as mean ± standard deviation (SD) of three independent experiments. For each experiment, concentrations were tested in triplicate in a 96-well plate. For anti-plasmodial and cytotoxicity activities the 50% inhibitory concentration (IC_50_) was calculated using nonlinear regression method. The model is an inhibitory sigmoid *E*max model [10]. Student-test was used to compare data at 95% confidence limit.

## Results

### Plant extraction

Methanolic extract and fractions of *I. senegalensis *were tested *in vitro *on *P. falciparum *chloroquine-sensitive (3D7) and chloroquine-resistant strains (7G8) for their anti-plasmodial activity. The yield is calculated as being the ratio of the mass of the extract on that of the starting dried powder. Thus, after methanolic extraction, freeze drying and partition, the yields obtained were (5.6 ± 0.75) %, (2.9 ± 0.8) % and (0.6 ± 0.2) % respectively for IM, IMW and IMD. After fractionation of IMD on Sephadex LH-20^® ^column, subfractions Da, Db and Dc represented respectively 20.5%, 14.6% and 60.2% of IMD. Approximately, 4% of products were lost during evaporation and freeze-drying process of subfractions.

### Haemolytic activity and cytotoxicity of extracts

Methanolic extracts and their fractions of *I. senegalensis *were evaluated for their haemolytic activity. Haemolytic effect observed with these extracts was always below the limit of detection and the same as the one observed with the solvent (MCM) which is considered in this test as a negative control; whereas the maximum haemolytic effect (100%) was observed with SDS (5% m:v) considered as positive control.

The cytotoxicity evaluated on Hepa 1-6 cells revealed IC_50 _as high as 133 ± 20 μg/mL, 122 ± 4 μg/mL and 447 ± 6 μg/mL for IM, IMD and IMW. Also no toxicity was observed on NHDF cells with extracts even at concentrations as high as 500 μg/mL. Table 1 summarizes the cytotoxicity against cells used as well as selectivity indexes of the plant extracts which remained above 35 whatever the extract sample was.

**Table 1:** Anti-plasmodial activity, cytotoxicity and selectivity indexes of extracts IM, IMD and IMW

Extracts	**IC**_**50 **_± SD* (μg/mL) for *P. falciparum *strains		**IC**_**50 (Hepa 1-6**_^**+**^_**)**_**(μg/mL)**	**IC**_**50 (NHDF**_^**+**^_**)**_**(μg/mL)**	SI
				
	3D7	7G8			
IM	4.7 ± 0.2	8 ± 1	133 ± 20	> 500	> 106

IMD	0.9 ± 0.2	4.1 ± 0.1	122 ± 4	> 500	> 500

IMW	14.2 ± 0.7	32 ± 2	447 ± 16	> 500	> 35

Chloroquine (nM)	44 ± 1	658 ± 14	> 800	> 800	> 18

* Standard deviation

+NHDF: Normal Human Dermal Fibroblasts

+Hepa 1-6: Mouse hepatocytes

IM: Methanol extract of *I. senegalensis *leaf extract, IMD: Apolar fraction of IM

IMW: Polar fraction of IM, SI: selectivity index.

### *In vitro Plasmodium falciparum *growth inhibition

The *P. falciparum *3D7 (chloroquine-sensitive) and 7G8 (chloroquine-resistant) strains used gave, with chloroquine, IC_50s _of 44 ± 1 nM and 658 ± 14 nM respectively. No measurable effect on parasite survival was observed in MCM containing DMSO at 0.5% and even with the freeze-dried methanolic extract *of Khaya senegalensis *stem bark which IC_50 _on 3D7 strain was higher than the highest concentration investigated (100 μg/mL). Anti-plasmodial activities of extract, fractions and subfractions are presented in Tables 1 and 2.

**Table 2:** Anti-plasmodial activity of fractions Da, Db and Dc

Fractions from IMD	IC_50 _± SD (μg/mL) for *P. falciparum *strains	
	
	3D7	7G8
Da	3.2 ± 0.2	1.7 ± 0.1

Db	2.56 ± 0.02	0.94 ± 0.06

Dc	4.2 ± 0.08	4.1 ± 0.1

Da, Db and Dc are fractions obtained after separation of IMD on a Sephadex^® ^LH-20 column.

Da, Db and Dc are fractions obtained after separation of IMD on a Sephadex LH-20^® ^column.

**Figure 3:**
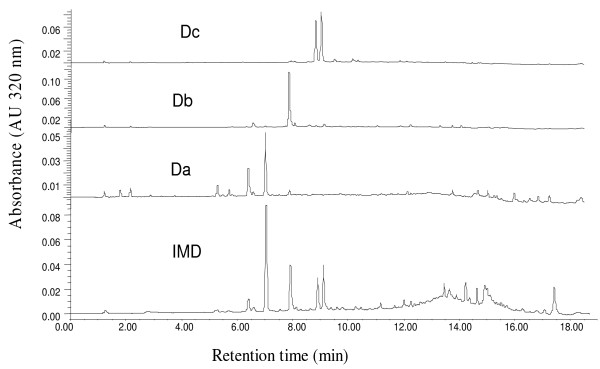
**Chromatographic profiles of IMD and subfractions Da, Db et Dc in similar conditions**.

Fraction IMD and subfraction Db of *I. senegalensis *A. Juss. leaves extract exhibited the highest inhibition with IC_50 _of 4.1 ± 0.1 μg/mL and 0.94 ± 0.06 μg/mL on *P. falciparum *7G8 strain (chloroquine-resistant). Their chromatographic profiles are presented in Figure 3.

## Discussion

Indigenous to western and central Africa, *I. senegalensis *is found growing wild on light sandy soils in the savannah areas of Senegal, Gambia, Northern Ghana, Guinea, Nigeria and parts of Sudan [20]. In Senegal, *I. senegalensis *mature leaves powdered are decocted and prescribed by healers against malaria and fevers. About 50 g of dried leaves are boiled in 1 L of water during 1 to 2 h. Leaves decoction is often mixed with honey to ovoid unpleasant taste. A posology of a cup of coffee (50 mL) three times per day for adults usually for 7 days is usually prescribed.

A bibliographic research related to the bioactivity of *I. senegalensis *revealed only antihyperglycemic activity of the leaves ethanolic extract [21]. Also a phytochemical study done on the plant tuber revealed the presence of icacenone (0.08%), icacinol (0.03%), β-sitosterol (55%), stigmasterol (45%) and hardwikiol linoleate [22,23].

Because *I.senegalensis *is traditionally taken against fevers and malaria after decoction with water, methanol, a polar solvent was used for plant extraction. However, it is so important to make sure that the use of the drug does not present toxic effects for human health. Thus, before starting *in vitro *evaluation of *P. falciparum *susceptibility to natural extract, the haemolytic activity of the extracts was evaluated on red blood cells with high concentrations exceeding those used in this work and reaching 100 μg/mL. A preliminary investigation of the freeze dried methanolic extract toxicity of *I. senegalensis *revealed no haemolytic effect *in vitro *on red blood cells. These first results, which were not amount-dependant, tended to confirm the traditional use by oral way of the decoctions and macerations of these natural extracts by the autochtone populations with a long history of use and without side effects. The haemolytic activity was of the same order of magnitude as those of negative control and chloroquine, a pure well tolerated molecule.

Because metabolism of xenobiotic taken by oral route as *I. senegalensis *leaf extract take place principally in the liver, it appeared important to evaluate the cytotoxic activity of active extracts on mouse hepatic cells (Hepa 1-6). The cytotoxicity test on Hepa 1-6 cell line revealed IC_50 _> 100 μg/mL for methanolic extract from *I. senegalensis *leaf and all obtained fractions (Table 1). As well, no toxicity was observed on NHDF cells (IC_50 _> 500 μg/mL). It is generally considered that pharmacological efficacy is not due to *in vitro *cytotoxicity when selectivity index (SI) >10 [24,25]. Consequently these extracts can be considered no toxic since all calculated selectivity indexes are higher than 35 (Table 1). This let us believe that activity obtained with *I. senegalensis *leaves extracts is not due to general toxicity against *P. falciparum *but can be explained by specific anti-plasmodial activity. These high selectivity indexes enabled further investigations on extracts and should offer the potential for safer therapy.

This *in vitro *evaluation of anti-plasmodial activity of *I. senegalensis *leaf extracts, showed that IC_50 _of IM, IMD and IMW were respectively 4.7 ± 0.2 μg/mL, 0.9 ± 0.2 μg/mL and 14.2 ± 0.7 μg/mL on *P. falciparum *chloroquine-sensitive 3D7 strain, and 8 ± 1 μg/mL, 4.1 ± 0.1 μg/mL and 32 ± 2 μg/mL on chloroquine-resistant *P. falciparum *7G8 strain (Table 1).

Recently, stringent endpoint criteria were set for anti-plasmodial activity, taking selectivity in account. Then relevant activity relates IC_50 _values below 100 μg/mL for extracts [26] and below 25 μM for pure compounds [27]. In this work, it is observed that all extracts gave an IC_50 _below 20 μg/mL (Table 1). This can be explained by non exhaustive and specific partition of active compound(s) between polar and apolar solvents and/or with presence of different active compounds in *I. senegalensis *leaf extract. So the active molecule(s) could be present in variable amount in both three extracts IM, IMD and IMW. The extract IM showed good anti-plasmodial activity but was less active on 3D7 and 7G8 strains than the defatted extract and partitioned extract IMD. This results can be explained by the fact that IM not defatted with pentane contained many pentane soluble fatty impurities which may reduce the anti-plasmodial activity and emphasize on the importance of extraction and purification protocols to confirm traditional use of plants. The less active extract was IMW which is the most polar one. Nevertheless, it was noted that the most active extract (IMD) gave an IC_50 _below 5 μg/mL (4.1 ± 0.1 μg/mL) on *P. falciparum *7G8 strain and below 1 μg/mL on *P. falciparum *3D7 strain. This extract showed predominant compounds in its chromatographic profile (Figure 3).

With the aim to find active subfractions and consequently identify the active substance(s) on *P. falciparum*, IMD was fractionated on a Sephadex LH-20^® ^column eluted by mixture of MeOH-H_2_0 (80:20, v/v) to produce three subfractions Da, Db and Dc. These subfractions contain compounds which are chemically different as shown by the chromatographic profiles (Figure 3). Table 2 shows very active subfractions with IC_50 _< 5 μg/mL. Nevertheless Db is significantly the most active one on both 3D7 and 7G8 strains with IC_50 _respectively of 2.56 ± 0.02 μg/mL and 0.94 ± 0.06 μg/mL (p < 0.05).

The IC_50 _of Dc is equal to that of IMD on *P. falciparum *7G8 strain. This means that Dc explains alone the activity of IMD and there is no need for Da and Db to explain the activity of IMD. This observation can be explained, taking into account the yields obtained after chromatographic separation. The amount of Dc present in IMD is 4 and 3 fold higher than those of Db and Da. With the hypothesis that Da, Db and Dc inhibit *P. falciparum *7G8 strain growth by a similar mechanism implying the same sites of action, Dc action is predominant in IMD. Then Dc will saturate the sites of action and there is no need of Da and Dc to justify the activity of IMD. Nevertheless, when tested separately, Da and Db can act on the *Plasmodium *without any competition on the same sites of action and showed higher anti-plasmodial activity (1.7 μg/mL and 0.8 μg/mL) than Dc (4.1 μg/mL) on the chloroquine-resistant strain 7G8. Considering the IC_50 _values obtained on this strain, it appears that the anti-plasmodial activity of Dc is 4.5 fold and 2.4 fold lower than those of Db and Da. One can see that these anti-plasmodial activities are correlated with the yields obtained after fractionation. The best anti-plasmodial activity obtained with Db on the chloroquine-resistant strain justifies our choice to prioritize this subfraction for further investigations.

Concerning the chloroquine-sensitive strain 3D7, it appears that the subfractions act by a different mechanism than that used on the chloroquine-resistant strain. Da, Db and Dc seem to act by different sites of action. Then their activities are additive in IMD which gave a best anti-plasmodial activity on the 3D7 strain (0.9 μg/mL). The fractionation does not give better anti-plasmodial activity than IMD and appears not useful concerning the 3D7 strain.

The fact that IMD extract present surprisingly higher activity compared to the ones of Da, Db and Dc (on the 3D7 strain only) can be explained mainly by a synergistic activity and incidentally by the loss of synergizing (or active) components during the process of fractionation. Indeed fine fractionation can lead to loss of activity due to decomposition or transformation of active constituents to less active substances (oxidation of phenolic compounds for example). Many plant extracts contain antioxidant compounds, which might protect labile substances in a crude extract but, upon fractionation such protective substances might become separated from the susceptible compounds, so that the later are quickly oxidised [28].

The IC_50 _of Db on *P. falciparum *7G8 strain (0.94 μg/mL) was lower than that on *P. falciparum *3D7 strain. This shows that there is no correlation between IC_50 _value obtained with Db and the chloroquine-sensitivity of the strain tested. This phenomenon was described by several authors testing anti-plasmodial activity of natural products [29-31].

Theses IC_50 _values seemed sufficiently potent to warrant further biological investigation on Db and to identify active principle(s).

According to the WHO recommandations and previous works [32-34], anti-plasmodial activities of plant extracts were classified as follows: highly active extracts with IC_50 _< 5 μg/mL, promising activity at 5-15 μg/mL, moderate activity at 15-50 μg/mL and inactivity at > 50 μg/mL.

Many plants belonging to other traditional medicines gave active extracts and very active purified molecules against *P. falciparum *strains. Nevertheless, the results of this study can be compared to those of known anti-plasmodial plants such as *Nauclea latifolia*, the stem bark's decoction of which not purified gave an IC_50 _of 1.7 ± 0.3 μg/mL on Nigerian chloroquine-sensitive strain (IC_50 _for chloroquine: 35 ng/mL) [35]. Plants considered as reference drugs by numerous authors because of their wide use in traditional medicine, have IC_50_s against chloroquine-resistant strains of 3.9 μg/mL (*Artemisia annua*) [36], 2.35-12.6 μg/mL (*Azadirachta indica*) [37] and 2.2 ± 0.4 μg/mL (*Nauclea latifolia*) [35] in similar experimental conditions. Compared to the results obtained with the Db fraction on chloroquine-resistant 7G8 strain (0.94 μg/mL), *I. senegalensis *shows an anti-malarial potential which can be revealed by a bioguided fractionation and a fine isolation of the active organic compounds.

It is the first scientific demonstration that *I. senegalensis *leaves extracts inhibit *P. falciparum *strains 3D7 and 7G8 growth at low concentration far below 5 μg/mL, and with satisfactory selectivity indexes. The selectivity indexes higher than 10 suggest that the anti-plasmodial activity of *I. senegalensis *is not due to general toxicity but can be explained by specific activity.

## Conclusion

The results achieved with this work constitute a proof that *I. senegalensis*, discovered by an ethnopharmacological survey, is a promising plant with regard to anti-malarial phytotherapy research and support continuous investigation of natural resources to discover new anti-malarials. Although these results add interesting informations to malaria research, the present study is preliminary. The next step will consist to identify the active compound (s) of *I. senegalensis*, to synthesize analogues and to run *in vivo *studies.

## Competing interests

The authors declare that they have no competing interests.

## Authors' contributions

SOS (PhD student) contributed in collecting plant sample and identification, confection of herbarium, running the laboratory work, analysis of the data and drafted the paper. SP contributed to biological studies. IF contributed in plant identification and herbarium confection. MZ contributed to chromatographic analysis. SE contributed to critical reading of the manuscript.

YMD contributed to plant collection. EM and EC designed the study, supervised the laboratory work and contributed to critical reading of the manuscript. All the authors have read the final manuscript and approved the submission.
